# Downregulation of IRF7-mediated type-I interferon response by *LmCen*^*–/–*^ parasites is necessary for protective immunity

**DOI:** 10.1038/s41541-024-01032-6

**Published:** 2024-12-19

**Authors:** Telly Sepahpour, Jalal Alshaweesh, Nazli Azodi, Komudi Singh, Derek D. C. Ireland, Farzaneh Valanezhad, Risa Nakamura, Abhay R. Satoskar, Ranadhir Dey, Shinjiro Hamano, Hira L. Nakhasi, Sreenivas Gannavaram

**Affiliations:** 1 Division of Emerging and Transfusion Transmitted Diseases, CBER, FDA, Silver Spring, MD 20993 USA; 2 Department of Parasitology, Institute of Tropical Medicine (NEKKEN), The Joint Usage/Research Center on Tropical Disease, Nagasaki University, Nagasaki, Japan, and Graduate School of Biomedical Sciences, Doctoral Leadership Program, Nagasaki University, Nagasaki, Japan; 3 National Heart Lung Blood Institute (NHLBI), NIH, Bethesda, MD USA; 4 Office of Pharmaceutical Quality, Center for Drug Evaluation and Research, US Food and Drug Administration, Silver Spring, MD USA; 5 Department of Pathology and Microbiology, Ohio State University, Columbus, OH USA

**Keywords:** Live attenuated vaccines, Parasitic infection

## Abstract

Leishmaniasis is a tropical disease caused by *Leishmania* parasites and currently has no licensed vaccines. We developed a dermotropic *Leishmania major* centrin gene-deleted strain (*LmCen*^–*/*–^) as a live attenuated vaccine. Recent studies have shown that type I interferons (IFNs) play important roles in immunity to parasitic and viral pathogens. However, their relevance in protective immunity following vaccination is not understood. We found that immunization with *LmCen*^*–/–*^ induces a transient increase in type I IFN response along with its regulatory factor IRF7 that is downregulated 7–21 days post-immunization, coincided with the induction of a robust Th1 adaptive immune response. Challenge infection with virulent *L. donovani* parasites showed a significant reduction of splenic and hepatic parasite burden in IRF7^–/–^ mice than wild type mice following immunization with *LmCen*^*–/–*^, suggesting that ablation of type I IFN response is a pre-requisite for the induction of *LmCen*^*–/*–^ mediated Th1 immunity against *L. donovani* infection.

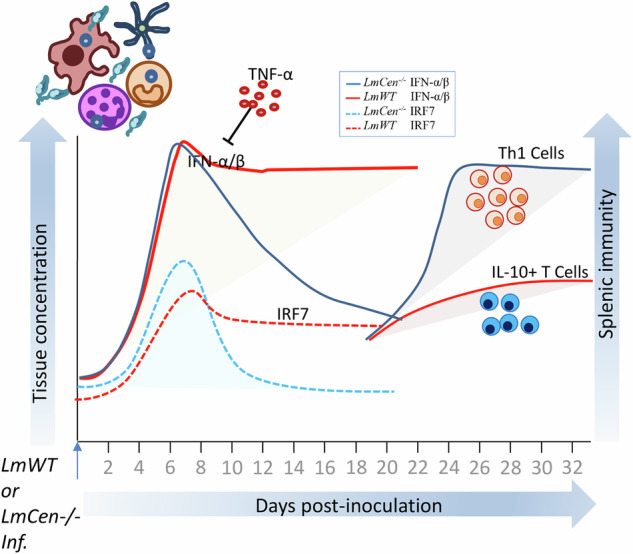

## Introduction

Leishmaniasis is a neglected tropical disease prevalent in developing countries. The World Health Organization estimates that more than 1 billion individuals living in these endemic areas are at risk of infection^1^. and over 1 million new cases occur annually. *Leishmania* is mainly transmitted through the bite of the female sandfly and other modes of transmission such as organ transplantation and blood transfusions are also demonstrated^2^. Leishmaniasis may manifest as cutaneous (CL), mucocutaneous (MCL) or visceral (VL) depending upon the infecting *Leishmania* species^3^. CL is often a milder disease accompanied by self-healing skin ulcerations in most instances but leaves a scar after cure. VL is more serious and can cause death if not treated. There are several therapeutics available; however, they are often expensive, highly toxic, and require hospitalization. Historical reports of successful immunity against leishmaniasis described deliberate inoculation with a low dose of virulent *Leishmania major* parasites, termed “Leishmanization”^4^. Although this technique has proved successful in conferring protection, it is discontinued due to safety concerns. Currently there are no approved anti-*Leishmania* vaccines for human uses^5^. In our laboratory we developed a live attenuated vaccine using a *centrin* gene deleted strain of *L. major* (*LmCen*^−/−^) and showed protection against cutaneous and visceral leishmaniasis in pre-clinical studies^4,5^

Mammalian cells express different pattern-recognition receptors (PRRs) that can detect the pathogen-associated molecular patterns (PAMPs) of viruses and other microbes, which leads to the activation of anti-microbial responses^6^. Cellular receptors such as RIG-I and MDA5, NOD1/2 and toll like receptors (TLRs) recognize viral RNA or other ligands. Following activation of receptors like TLRs, the signaling cascades converge on a family of IFN-regulatory factors (IRFs) that mediate the production of type I interferons IFNα/β^7^. Mainly IRF-3 and IRF7 are shown to regulate IFNα/β production, although IRF1, IRF5 and IRF8 can also induce type-I responses^8^. IFN-α/β mediate the anti-viral response through IFN receptors IFNAR1 and IFNAR2^7^. The effects of IFNα/β are not limited to anti-viral response in an acute stage but affect various aspects of innate and adaptive immunity. For instance, type-I IFNs are involved in migration, activation and differentiation of DCs, and their ability to stimulate naive T cells^9–13^, Type I interferons (IFNs) can also affect CD4^+^, CD8^+^ T cells and NK cell responses depending on the pathogenic agent^7,14^, In studies of *Plasmodium berghei* infection using IFNAR1^−/−^ mouse models, type I IFN signaling is shown to directly affect DC function, limiting their ability to prime IFN-γ producing CD4^+^ T cells^15,16^, Studies in severe malaria showed the role of IRF-1 in regulating IL-12 and IFN-γ dependent control^17^. IFNα/β have been shown to regulate the production of nitric oxide, indicating a role for the type-I response in *L. major* parasite control in C57BL/6 mice^18^. Later studies showed that upon infection, metalloprotease gp63 from *L. major* parasites activates the translational repressor 4E-BP1 that presumably dampens type-I IFN production through the repression of IRF7 expression^19,20^, In contrast, IFN-β has been shown to impair the production of superoxide mediated parasite killing in *L. amazonensis* and *L. braziliensis* infections of human macrophages^21^. In studies with *L. donovani* parasites, type-I IFNs signaling is shown to be important for the development of polyclonal B cell activation and hypergammaglobulinemia during chronic infection and plays a harmful role in VL^22–25^. Similarly, IFN-α/β expression was elevated in acute VL cases compared to post-treatment and endemic controls, and IFNAR1^−/−^ mice controlled splenic *L. donovani* burden compared to C57BL/6 mice, suggesting that type-I IFN responses promote pathology^26^. Therefore, there seems to be variable roles of type-I immunity in *Leishmania* pathogenesis and can depend on the stage of infection i.e. early verses chronic stage.

The crosstalk between type-I and type-II IFN responses attracted significant attention in immuno-parasitology, since protozoan parasites are potent inducers of IFN-γ produced by T and NK cells^7^. In both *Plasmodium* and Lymphocyte Choriomeningitis Virus (LCMV) infections, reduced type-I response restored the ability of CD4^+^ T cells function and IFN-γ production^15,16,27^, High levels of IFN-β have been shown to induce progressive disease in both *L. major* and *L. donovani* models^25,28^ Accordingly, elevated type-I IFNs are shown to induce IL-10 producing Tr1 cells in *L. donovani* infection suggesting that blockade of IFN-α/β may provide therapeutic benefit against VL^26^. In studies with *L. donovani* parasites, IRF-5 has been shown to play a critical role in the development of T helper 1 responses^29^.

Of the several IRFs that regulate the production of type-I IFN response, IRF7 has been recognized as a master regulator^30^. IRF7 is constitutively expressed and acts in a positive feedback loop in the presence of type-I IFNs^31^. IRF7 can be activated following ligation of TLR3, TLR4 or TLR9. Phosphorylation and nuclear translocation of IRF7 is necessary for downstream transcriptional activities^31^. IRF7 is shown to be associated with the production of IL-12 upon infection of human DCs with *L. major* parasites^32^. Furthermore, IRF7 is necessary for control of *L. donovani* parasites in liver and spleens since hepatic T cells from IRF7^–/–^ mice produced significantly less IFN-γ upon PMA-ionomycin restimulation, and were deficient in NOS production^33,34^, CD4^+^ T cells from IRF7^–/–^ mice upon infection with *L. donovani* parasites produced IFN-γ levels comparable to wild type mice 14- and 28- days post-infection^29^.

As illustrated by these studies, IRF7 mediated innate and adaptive immunity affects the early and acute stage infection in *Leishmania*. However, its role in protective immunity induced by immunization has not been explored. In this study, we analyzed the gene expression after infection with virulent *L. major (LmWT)* compared to immunization with *centrin* gene-knockout *L. major (LmCen*^*–/–*^*)* parasites. An enrichment of type I IFN genes were shown as differentially expressed genes in mice infected with *LmCen*^*–/–*^ and *LmWT* parasites, which was accompanied by an elevated type-I IFNs in the sera. We explored the interplay between type-I IFNs and the development of host protective Th1 immunity in mice immunized with *LmCen*^*–/–*^ parasites and challenged with *L. donovani* parasites and showed that downregulation of IRF7 mediated production of IFN-α/β is critical for the induction of protective immunity.

## Results

### NanoString analysis identifies distinct transcriptional profiles at the site of inoculation of *LmCen*^–/–^ immunized mice

**Fig. 1:**
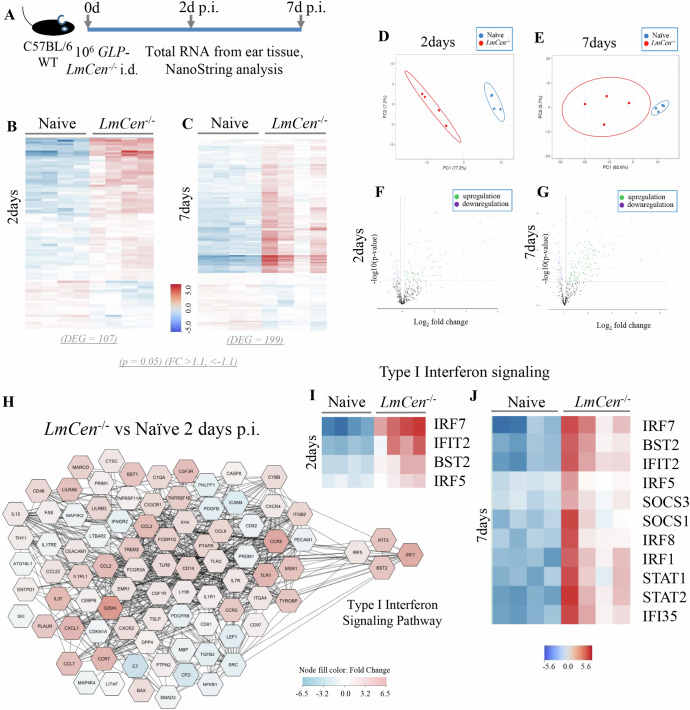
Distinct transcriptional profiles in tissue of *LmCen*^*−/−*^ immunized C57BL/6 mice. **A** Schematic representation of the study to identify differential expressed genes following immunization with *LmCen*^*–/–*^ parasites using the NanoString platform. Heatmaps showing distinct clustering of the genes indicated by red-blue colors representing the log2 (fold change) in *LmCen*^*−/−*^ vs Naïve at 2-days p.i. (**B**) and 7-days p.i. (**C**). Principal component analysis scatterplot of normalized data at 2 days (**D**) and 7 days (**E**) p.i. Data from naïve set are shown in blue and data from *LmCen*^*−/−*^ set are shown in red. Values for each group are bound by an eclipse. Volcano plots of *LmCen*^*−/−*^ vs Naïve at 2-days (**F**) and 7-days (**G**), representing genes detected above background levels under each condition, are shown in black. Significantly upregulated genes are green, and significantly downregulated genes are purple. The x-axis represents the log ratio of the fold change, and the y-axis is the negative log of p-adj/*p* value. p.i. post immunization. **H** The gene network through GO enrichment analysis of *LmCen*^*–/–*^ immunized vs Naïve at 2 days post immunization showing the upregulation of transcripts associated with the IRF-7 mediated type-I IFN response in the ear tissue of *LmCen*^*–/–*^ immunized mice. Gene Set Analysis (GSA) derived from heatmap clustering showing the expression of type-I IFN associated elements on (**I**) 2 days and (**J**) 7 days post-immunization.

To identify the early immune mechanisms antecedent to vaccine-induced protection, mice were immunized with *LmCen*^–/–^ or infected with virulent *L. major*. RNA was isolated from the site of inoculation (ear tissue) and analyzed by NanoString (nCounter Immunology Mouse Panel) at 2-, and 7-days post-infection (Fig. 1A; Supplementary Fig. 1A–C). The transcriptomic analysis showed clusters of genes either up- or down-regulated in *LmCen*^–/–^ immunized mice compared to naïve controls at 2 days post-immunization (p.i.) (Fig. 1B) and at 7 days p.i. (Fig. 1C). The principal component analysis (PCA) of the statistically significant genes at both 2 days (Fig. 1D) and 7 days p.i. (Fig. 1E) showed distinct clustering between naïve and *LmCen*^–/–^ groups. The PCA plots of *LmCen*^*–/–*^ vs naïve at 2 days p.i. (Fig. 1D) through X and Y axis showed PC 1 and PC 2 explaining 77.2 and 7.2% of the total variance, respectively. Similarly, *LmCen*^*–/–*^ vs naïve at 7 days p.i. (Fig. 1E) demonstrates X and Y axis showing PC 1 and 2 that explain 82.6 and 5.7% of the total variance, respectively. The volcano plots with a fold change threshold of ±1.1 and a *p* value threshold of 0.05 were used to identify the significantly up- or down-regulated genes in *LmCen*^−/−^ immunized group (Fig. 1F, G). A total of 79 genes were upregulated and 28 were downregulated and recorded as differentially expressed genes (DEGs) between the *LmCen*^−/−^ and naïve controls at 2 days p.i. out of the 547 genes on the NanoString nCounter Mouse Immunology Panel (Fig. 1F, Supplementary Table 1). Similar analysis at 7 days p.i. showed 140 upregulated and 59 downregulated DEGs between the *LmCen*^–/–^ and naïve controls (Fig. 1G, Supplementary Table 1). The DEGs identified in *LmCen*^–/–^ immunized mice via the volcano plots (Fig. 1F, G) were used to perform GO enrichment inquiry and to create an integrative gene network analysis of *LmCen*^*–/–*^ immunized vs naïve groups to better visualize the alterations at the transcriptome and associated pathways detected in our dataset. Similar analysis identified the enrichment of IRF7 mediated type-I IFN response in both *LmCen*^*–/–*^ immunized and *LmWT* infected mice at 2 days (Fig. 1H) and 7 days post immunization (Supplementary Fig. 1). Transcripts associated with type-I IFN pathway showed that enrichment of IRF7 occurred in both *LmWT* and *LmCen*^*–/–*^ infections (Supplementary Fig. 1). Gene set analysis-derived heat map showed four (Fig. 1I) and eleven genes (Fig. 1J) associated with type-I IFN response to be upregulated in *LmCen*^−/−^ immunized mice at the 2- and 7-day time-points, respectively.

### Distinct expression of type-I interferon observed in mice following *LmCen*^*–/–*^ and *LmWT* infections

**Fig. 2:**
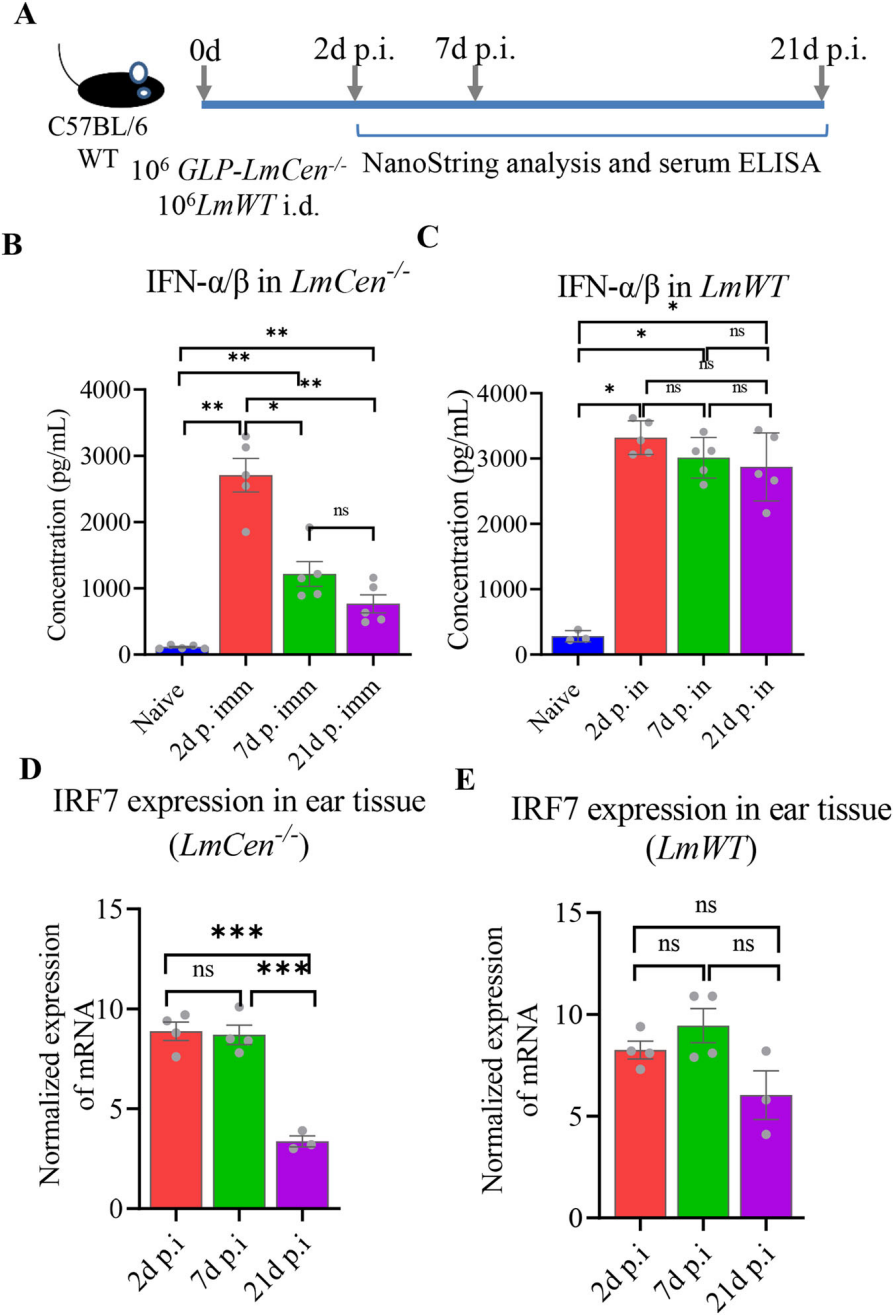
Distinct expression of type-I interferon response observed in C57BL/6 mice immunized with *LmCen*^*–/–*^ parasites. **A** Schematic representation of the study to measure the kinetics of type-I Interferon protein expression in sera and IRF-7 expression in the ear tissue following *LmCen*^*–/–*^ immunization or infection with *LmWT* parasites. **B** IFN-α/β levels at 2-days, 7 days, and 21 days post immunization with *LmCen*^*–/–*^ (**C**) and with *LmWT* at the identical time points. **D** Normalized expression of IRF7 in the ear tissue as measured by NanoString analysis at 2 days, 7 days, and 21 days post immunization with *LmCen*^*–/–*^ (**E**) and infection with *LmWT* parasites. Type I Interferon values are shown as mean ± SEM representing one experiment and *p* value determined with Mann–Whitney two-tailed test, and IRF7 expression showing *p* value determined via unpaired *t* test.

To verify if the elevated expression of IRF7 gene(s) results in a corresponding production of IFN-α/β, we measured the kinetics of IRF7 expression and the type-I IFN response in ear tissues and sera in mice infected with *LmCen*^*–/–*^ or *LmWT* parasites, respectively (Fig. 2A). Following immunization with *LmCen*^*−/−*^, levels of IFN-α/β and mRNA expression of IRF7 were measured and compared to those in *LmWT* infection (Fig. 2B–G). The *LmCen*^–/–^ immunization induced the expression of IFN-α/β in sera, which started increasing by day 2 p.i. followed by a significant decline by day 7 p.i. through day 21 p.i. (Fig. 2B). Concomitantly, the expression of IRF7 in the ear tissues, the site of inoculation, was significantly elevated at 2 days p.i. and declined significantly by day 21 p.i. (Fig. 2D). In *LmWT* infected mice, the levels of IFN-α/β in sera also started increasing by day 2 p.i. and remained elevated even at day 21 p.i. (Fig. 2C). Correspondingly, the expression of IRF7 in the ear tissue of *LmWT* infected mice was elevated at 2 days p.i. and stayed elevated through 21 day p.i. (Fig. 2E), suggesting the association between the elevated expression of IRF7 and IFNα/β. Naive mice were injected with PBS to rule out the differences in IRF7 or IFN-α/β expression due to needle injury. Hence, the differences in IFN-α/β observed on 21 days post-inoculation between *LmCen-/-* immunization and WT infection could not be due to needle injury (Fig. 2B, C). To verify if the differential regulation of IFN-α/β production observed in mice inoculated *LmCen*^*–/–*^ and *LmWT* is specifically mediated by IRF7, we measured the expression of other IRFs such as IRF3 commonly linked to the type-I response and IRFs 1 and 8, linked to innate immune response. We did not detect IRF3 in either infection in the NanoString analysis. Meanwhile, in either *LmCen*^*–/–*^ or *LmWT* infected mice, the expression of IRFs 1 and 8 did not show any significant difference between 7 and 21 days p.i. (Supplementary Fig. 2A–D, respectively) whereas a small reduction was observed in *LmCen*^*−/−*^ immunized mice compared to naïve at the same time points (Supplementary Fig. 2D). In contrast, IRF-5 transcripts were detected in *LmCen*^*−/−*^ infection on days 2 and 7, and in *LmWT* infection on days 7 only. By 21 days post-infection IRF5 was not detected in both infections (Supplementary Fig. 2E, F).

### Absence of IRF7 signaling enhances Th1 immune response following immunization with *LmCen*^–/–^ parasites at day 21 post immunization

**Fig. 3:**
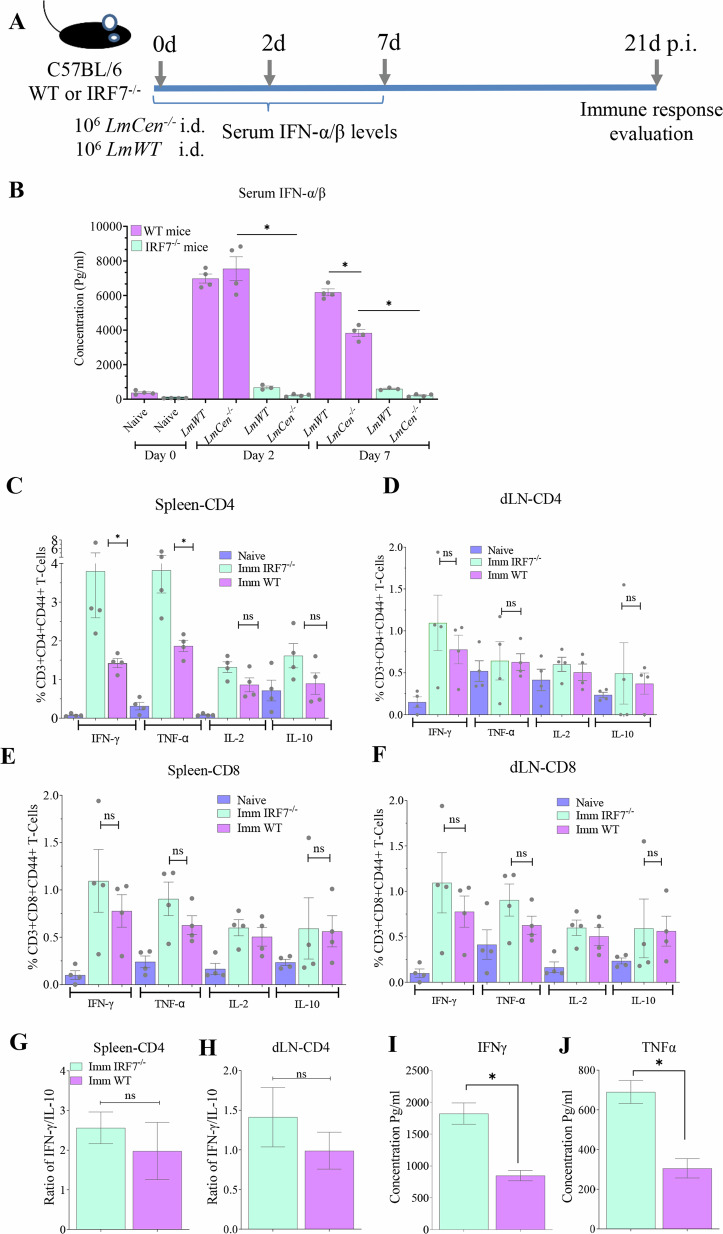
IRF7^–/–^ mice induced higher protective Th1 response than C57BL/6 WT mice following immunization with *LmCen*^*–/–*^ parasites. **A** Schematic representation of the experimental design conducted at the Nagasaki University. **B** Expression of IFN-α/β in the sera of mice infected with *LmWT* or *LmCen*^*–/–*^ was measured at days 0, 2 and 7 p.i. by ELISA in WT and IRF7^–/–^ mice. **C** The percentage of IFN-γ, TNF-α, IL-2, and IL-10 producing CD4 + T cells was measured following restimulation with Leishmania freeze thaw antigens in the splenocytes and (**D**) in draining lymph node cells at 21 days post-immunization. The percentage of the cytokine-producing CD8 + T cells isolated from the spleen (**E**) and draining lymph node cells (**F**). The corresponding ratio of IFN-γ/IL-10 produced by CD4 + T cells isolated from the spleen (**G**) or draining lymph nodes (**H**). The levels of (**I**)IFN-γ and (**J**) TNF-α produced by the isolated splenocytes after stimulation with L. donovani antigen for 72 h. Results are shown as mean ± SD representing one experiment and *p* value determined with Mann–Whitney two-tailed test.

To test if the downregulation of type-I IFN response coincides and is associated with the development of IFN-γ dominant protective Th1 immunity, at first, the development of adaptive immune response was analyzed in C57BL/6 WT mice immunized with *LmCen*^*–/–*^ parasites at the indicated time point (Fig. 3 and Supplementary Fig. 3). The levels of IFN-γ, TNF-α, IL-2, and IL-10 were analyzed in splenocytes and draining lymph node cells at 21 days p.i. by flowcytometry (Supplementary Fig. 3). The gating strategy for identifying specific cell populations is shown in Supplementary Fig. 4. Activated CD4^+^ T cells (CD3^+^CD4^+^CD44^+^ T cells) showed a significant increase in IFN-γ, TNF-α and IL-2 in both splenocytes (Supplementary Fig. 3B) and dLNs (Supplementary Fig. 3C) of the immunized mice compared to naïve mice. Elevated IFN-γ, TNF-α and IL-2 response was also observed in the activated CD8^+^ T cells (CD3^+^CD8^+^CD44^+^ T cells) isolated from the spleens (Supplementary Fig. 3D) and lymph nodes (Supplementary Fig. 3E) of immunized mice compared to naïve controls. An elevated expression of IL-10 was observed in activated CD4^+^ T cells in both splenocytes and dLNs (Supplementary Fig. 3B, C), and in activated CD8^+^ T cells in splenocytes and dLNs of the immunized mice (Supplementary Fig. 3D, E). To test the role of IRF7 mediated IFN-α/β response in the development of Th1 immunity, both WT and IRF7^–/–^ mice were immunized with *LmCen*^–/–^ parasites, and both type-I and type-II immune responses were analyzed at identical time points as before (Fig. 3A). WT mice produced significantly elevated levels of serum IFNα/β after infection with either *LmWT* or *LmCen*^*−/−*^ parasites. In contrast, IRF7^–/–^ mice produced only background level of type-I IFNs after infection with *LmWT* or *LmCen*^*–/–*^ parasites on days 0, 2 and 7 p.i. (Fig. 3B). Additionally, serum IFN-α/β levels augmented in WT mice were significantly downregulated by day 7 p. i. in *LmCen*^*–/–*^ infection compared to *LmWT* (Fig. 3B). Analysis of type-II-IFN responses at 21 days post-immunization with *LmCen*^*−/−*^ parasites revealed that IFN-γ and TNF-α producing activated CD4^+^ T cells (CD3^+^CD4^+^CD44^+^ T cells) were significantly higher in the spleen of IRF7^−/−^ mice than those of WT mice, while the percentage of the IL-2 and IL-10 producing CD4^+^ T cells in the spleen and dLN were similar (Fig. 3C, D). Meanwhile, IFN-γ, TNF-α, IL-2, and IL-10 producing activated CD8^+^ T cells (CD3^+^CD8^+^CD44^+^ T cells) in the spleen and dLNs were not altered in immunized IRF7^−/−^ and WT mice (Fig. 3E, F). A higher percentage of IFN-γ over IL-10 producing CD4^+^ T cells was observed in both spleen and dLNs of IRF7^–/–^ mice (Fig. 3G, H). Both IFN-γ and TNF levels were significantly higher in splenocytes of IRF7^−/−^ immunized mice, suggesting the absence of IRF7 mediated IFN-α/β expression enhances the Th1 mediated immune response (Fig. 3I–J). The gating strategy for identifying specific cell populations is shown in Supplementary Fig. 5.

### Lack of IRF7 signaling enhances IFN-γ+ production in CD4 T cells following challenge with virulent *L. donovani*

**Fig. 4:**
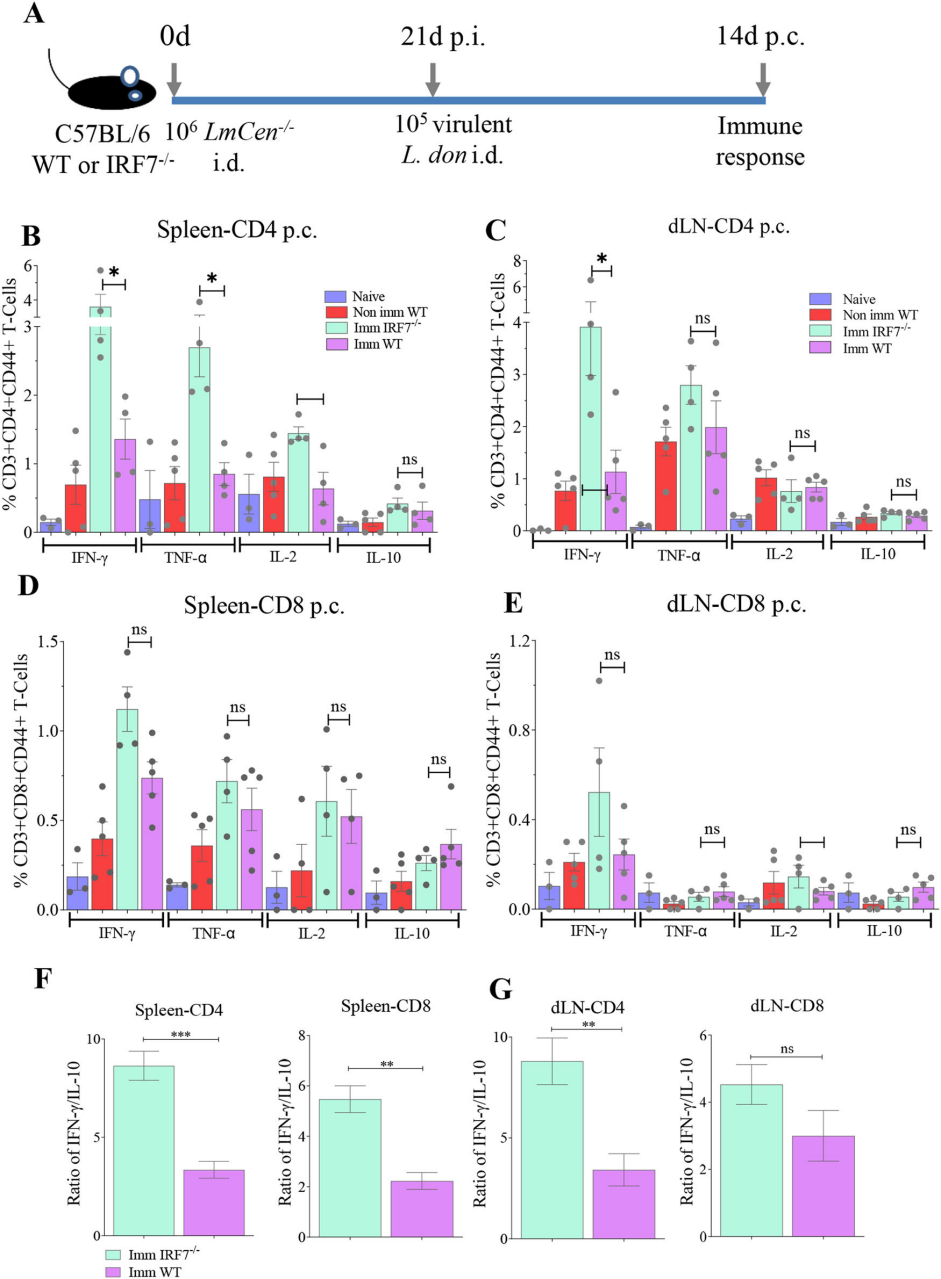
Lack of IRF7 signaling enhances the protective Th1 response following challenge with virulent *L. donovani* in *LmCen*^*−*/−^ immunized C57BL/6 mice*.* **A** Schematic representation of the experimental study conducted at the Nagasaki University. Percentage of IFN-γ, TNF-α, IL-2, and IL-10 producing T cells; activated CD4^+^T cells were isolated from the spleen (**B**) or draining lymph nodes (**C**) and simulated with *Leishmania donovani* (FTAg) at 14 days post-challenge. Similar responses from the activated CD8^+^ T cells isolated from the spleen (**D**) or draining lymph nodes at 14 days post-challenge. **E** The ratio of IFN-γ/IL-10 produced by activated CD4 ^+^ or CD8^+^ T cells from the spleen (**F**) and draining lymph nodes (**G**). Results are shown as mean ± SD and represent one experiment with the *p* values determined by Mann–Whitney two-tailed test.

To verify if the T cell response observed in IRF7^–/–^ mice following immunization with *LmCen*^–/–^ parasites results in acquiring protective immunity, WT and IRF7^–/–^ C57BL/6 mice were immunized and then challenged with virulent *L. donovani* strain (Fig. 4A). Immune response was analyzed by flowcytometry at 14 days post challenge in WT and IRF7^–/–^ mice (Fig. 4, Supplementary Fig. 6). Splenocytes isolated from non-immunized-challenged and immunized-challenged WT mice were stimulated with *L. donovani* freeze-thaw antigen (FTAg), and the production of IFN-γ, TNF-α, IL-2, and IL-10 was measured (Supplementary Fig. 6). Activated CD4^+^ T cells (CD3^+^CD4^+^CD44^+^ T cells) in the spleen of immunized WT mice produced significantly higher levels of IFN-γ, TNF-α, and IL-2, but not IL-10 compared to non-immunized WT mice (Supplementary Fig. 6B). An increase in IFN-γ and IL-10 producing activated CD4^+^ T cells was observed in the draining lymph nodes of immunized mice compared to non-immunized controls (Supplementary Fig. 6C). The IL-2 and TNF-α producing activated CD4^+^ T cells were similar in these groups (Supplementary Fig. 6C). The proportion of IFN-γ, TNF-α, IL-2 producing activated CD8^+^ T cells (CD3^+^CD8^+^CD44^+^ T cells) were significantly higher in the spleens and dLNs of immunized mice than non-immunized-challenged mice (Supplementary Fig. 6D-E). Significant difference in IL-10 producing activated CD8^+^ T cells was observed only in the spleen (Supplementary Fig. 6D) and not in dLN (Supplementary Fig. 6E). A higher ( > 2) ratio of IFN-γ over IL-10 was observed only in CD4^+^ T cells isolated from spleen of immunized mice (Supplementary Fig. 6F), but not in the dLNs or in CD8^+^ T cells (Supplementary Fig. 6G–I) suggesting a Th1 dominated immune response in immunized mice.

We next evaluated the impact of IRF7 signaling deficiency on the development of Th1 immune response by comparing the cytokine production in WT and IRF7^−/−^ mice following the challenge infection with virulent *L. donovani* parasites (Fig. 4A). A higher percentage of IFN-γ, TNF-α, and IL-2 producing activated CD4^+^ T cells were observed in the spleen of IRF7^–/–^ mice than WT mice (Fig. 4B). In the dLNs, IRF7^–/–^ mice showed similar increase in the IFN-γ producing CD4^+^ T cells (Fig. 4C). Both in the spleens and dLNs, the percentage of IL-10 producing CD4^+^ T cells were comparable between IRF7^−/−^ and WT mice (Fig. 4B, C). No significant differences were observed in the proportion of IFN-γ, TNF-α, IL-2, and IL-10 producing activated CD8^+^ T cells between the immunized IRF7^–/–^ and WT mice (Fig. 4D, E). However, a higher ratio of IFN-γ over IL-10 was observed in CD4^+^ and CD8^+^ T cells isolated from both the spleen and the dLN of the immunized IRF7^–/–^ mice than WT mice (Fig. 4F, G). Taken together, these data suggest that the lack of IRF7 signaling enhances the protective Th1 immune response of the immunized mice following the challenge with the virulent *L. donovani* parasites.

### *LmCen*^*−/−*^ immunization reduces the parasite burden in both WT and IRF7^–/–^ mice

**Fig. 5:**
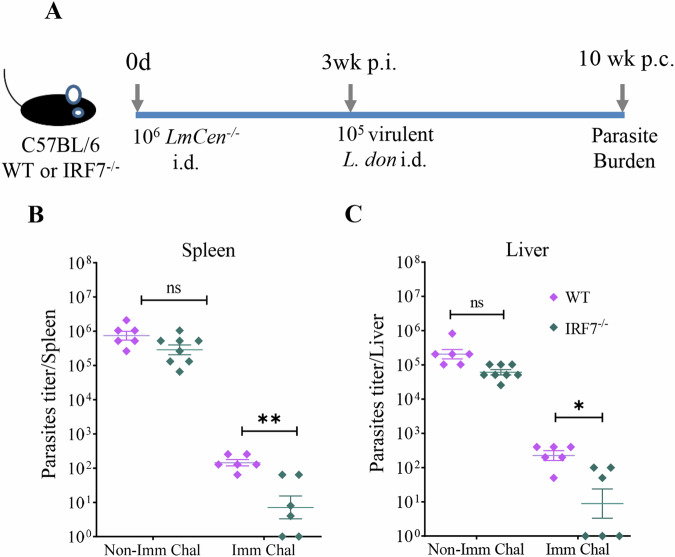
Splenic and hepatic parasite burden in control and IRF7^−/−^ mice. **A** Schematic representation of the experimental study. **B** Splenic and, **C** liver parasite burden from immunized Challenged IRF7^–/–^ and C57BL/6 WT mice. Results are representative of two separate experiments, shown as mean ± SEM and the *p* value was determined by Mann–Whitney two-tailed test.

To confirm whether the elevated IFN-γ production by CD4 T cells observed in mice immunized with *LmCen*^–/–^ parasites confers protection against virulent challenge, parasite burdens in spleen and liver tissues were determined by limiting dilution at 10 weeks post-challenge in WT and IRF7^–/–^ mice (Fig. 5A, Supplementary Fig. 7). There was significant reduction of splenic parasite burden in both IRF7^–/–^ and WT immunized mice compared to non-immunized mice (Fig. 5B, C and Supplementary Fig. 7B showing splenic parasite burden 35 days post-challenge) respectively. More importantly, IRF7^–/–^ mice immunized with *LmCen*^*−/−*^ parasites controlled splenic and hepatic parasite burdens significantly better than the WT mice following virulent *L. donovani* challenge (Fig. 5B, C). These results demonstrate that the enhanced Th1 immune response consists of the protective immunity after immunization with *LmCen*^–/–^ in WT and IRF7^–/–^ mice.

## Discussion

In this study, we have shown that IRF7 mediated regulation of type-I IFN response is a critical determinant of protective immunity induced by immunization with live attenuated *Leishmania* (*LmCen*^*–/–*^) parasites. Following ligation of pattern recognition receptors (PRR) such as TLRs by microbial products, IFN-α/β production is initiated through the activities of the IFN-regulatory factor (IRF) family of transcription factors^7^. Consistent with this understanding, >100 genes were found to be induced 2 days post-immunization in our study, which were associated with interferon signaling pathways. This expression peaked at 7 days post-immunization with 199 genes, while type I-IFN associated genes and signaling pathways continued though 21 days post-immunization. Additionally, we observed distinct transcriptional differences between *LmCen*^*–/–*^ and *LmWT* by 7 days post-inoculation. These differences observed in type-I IFNs could not have arisen due to the intrinsic differences in the proliferation of *LmWT* and *LmCen*^*–/–*^ parasites since we have observed no differences in parasite burdens at day 7 post-inoculation in the ear tissues in a separate study^35^.

Previous studies have established the role of type-I IRFs in innate immunity to viruses. In most viral infections, IRF-3 and IRF7 are considered fundamental IRFs that act in a two-step positive feedback loop to transcribe IFNα/β genes^36^. IRF-1/5/8 have also been linked to the induction of IFNα/β^31^. In the West Nile Virus infection, type-I IFNs are produced through the coordinated activities of IRFs 3/5/7^37^ whereas IRF-1 was critical for such response in Dengue virus infection^38^, indicating pathogen specific activation and/or requirement of various IRFs. In contrast to their critical role in viral clearance^39^, type-I IFNs exhibit host-protective or pathogenic functions in parasitic infections^20^. IFN-β and its downstream IL-10 response suppressed IFN-γ production by macrophages in *M. leprae* infection that causes progressive lepromatous lesions showing the inverse correlation between the two types of IFNs^40^. Induction of strong type-I IFN in *M. tuberculosis* in murine models either by agonists such as Poly-ICLC or by influenza infection resulted in greater susceptibility and impaired immunity by limiting the IFN-γ mediated microbicidal activities of macrophages^41^. Similar crosstalk between type-I and type-II IFNs has been studied in several parasitic infections including *Leishmania*, *Plasmodium* and *T. cruzi*^20^. IFN-α/β and NOS-2 are shown to play a protective role during the first few days of *L. major* infection^18^, whereas type I IFNs induced by endogenous viruses present in *L. guyanensis* suppressed the immune responses in this infection model^42^. In contrast, blocking type-I IFN responses led to limited recruitment of inflammatory monocytes and attenuated pathology in murine *L. amazonensis* infection^43^ suggesting that type-I IFNs may play distinct roles in different species of *Leishmania* parasites.

Distinct from their role in early stage of infection, type I interferons are emerging as key drivers of inflammation and immunosuppression in chronic infection. During persistent viral infection, type-I IFNs orchestrate a dysregulated immune response that leads to disease progression in LCMV, where blocking type-I IFN signaling enhanced virus specific CD4 T cells and resulted in viral clearance^44^. Interestingly, treatment with α-IFNR1 antibody accelerated viral clearance concomitant with a reduction in IRF-7 expression suggesting that in chronic LCMV models type-I IFNs are regulated by IRF-7^38^. Chronic virus infections are characterized by mutually antagonistic immunosuppression and inflammation. Recent evidence indicates that regulation of these immune states is integrally linked to type-I IFNs that induce many of the suppressive factors that limit immunity and promote chronicity^45^. Gene expression patterns between active and latent tuberculosis infections revealed that type-I IFNs promote chronicity^46^. Sustained production of type-I IFNs observed in *LmWT* infection indicates a similar immune-regulatory role in promoting a chronic infection. In contrast, transient induction of type-I IFNs in *LmCen*^*−/−*^ that was downregulated by day 21 post-immunization revealed a new immune regulatory mechanism in vaccine immunity. Supporting this hypothesis, controlled human infection model (CHIM) studies using *P. falciparum* showed that type-I IFNs suppressed innate immune cell function, IFN-γ production by parasitic-specific CD4^+^ T cells and promoted the development of IL-10 producing parasitic-specific IL-10-producing Th1 (Tr1) cells^47^. In contrast to viral infections, the role of type-I IFNs among most parasitic infections is investigated using IFNAR^−/−^ mouse models^16,18,48–50^, The role of various IRFs that is critical for the IFN-α/β production, and their impact on host adaptive immunity remain unknown. Early induction of IFN-α/β in *L donovani* infections are detrimental to the host by suppressing the CD4^+^ T cell dependent parasite control and skewing the CD4^+^ T cell response towards a pathology promoting IL-10^+^ Treg cells^16,26,51^, Blocking the type-I IFN signaling in IFNAR-1^−/−^ mice led to the control of *L. donovani* infection in liver and spleen, revealing that dampening type-I IFN response is essential for the establishment of host protective Th1 immunity. Similarly, our data on live attenuated *Leishmania* parasites, indicates that a down regulation of type-I IFN response results preceded the development of host protection mediated by IFN-γ producing CD4^+^ T cells. The role of IFN-γ producing CD4^+^ T cells in host protection analogous to leishmanization was previously shown by us^4,5^.

A substantial body of literature has established the protective role of CD4^+^ T cells in experimental cutaneous and visceral leishmaniasis models. A strong Th1 immune response to *L. major* parasites, mediates IFN-γ directed parasite control by activating macrophages leading to nitric oxide production and parasite elimination^52^.

Despite the importance of type-I IFN response that emerges early in the infection, the interplay between type-I IFN response and Th1 immunity has not been established in parasitic vaccines. Results from our current study using differential gene expression clearly indicated that IRF7 mediated type-I responses are down regulated by day 21 of immunization, coinciding with the emergence of potent Th1 responses^53^. In contrast, virulent infection with *L. major* parasites did not show similar reduction in both IRF7 expression and the IFNα/β levels, indicating that IRF7 mediated type-I IFN response plays a critical role in determining pathology in virulent infection and a protective response in the case of vaccination. In studies of blood stage *Plasmodium* infection, IRF7 was found not to be essential for tissue accumulation of parasites, cerebral symptoms, or brain pathology^54^. Blocking type-I IFN signaling by α-IFNAR1 mAb promoted fatal disease via IRF7-independent mechanisms. However, IRF7 significantly impaired early splenic Th1 responses and limited control of parasitemia during PbA infection^54^. In contrast, significant control of parasite burdens in spleen and liver upon *L. donovani* challenge in *LmCen*^*−/−*^ immunized IRF7^−/−^ C57BL/6 mice compared to WT C57Bl/6 mice suggests that IRF-7 mediated type-I IFNs negatively affect the protective immunity. Further, studies in IRF7^−/−^ mice^33^ showed that these mice carry significantly higher liver parasite burdens compared to wild type C57Bl/6 mice. Further studies in IFNAR1^−/−^ mice showed that these mice cannot spontaneously control parasite burden in the footpad, draining lymph node and spleen and carry comparable parasite loads in wild type mice over 150 days infection. However, upon immunization with CpG, mice lacking IFN-β were able to control the parasite burden in the foot pads and draining lymph nodes^55^, suggesting that lack of type-I IFN response does not lead to protective immunity.

The elevated levels of CD4^+^ Th1 immune response observed in IRF7^−/−^ mice following immunization with *LmCen*^*–/–*^ parasites and following challenge with virulent *L. donovani* validate this hypothesis. Several lines of evidence in our study indicate that the reduction of IFN-α/β levels in *LmCen*^*–/–*^ infection is mediated by the spontaneous reduction of IRF7 expression. The canonical models of type-I IFN production state that IRF3 is activated prior to IRF7 in viral infections that trigger TLRs or RIRs. In our analysis, we did not detect IRF3 in both *LmWT* and *LmCen*^*–/–*^ infections indicating that IRF7 alone may be sufficient to induce the type-I response following *Leishmania* infection. In addition to IRF7, our study also revealed significant expression of IRF1 and IRF8, however their expression did not strongly correlate with the robust induction of type-II IFN (IFN-γ) expression following immunization with *LmCen*^*–/–*^ parasites. Previous studies showed these transcriptional regulators was induced in human DCs following *L. major* infections, but not *L. donovani*.^56^. Additionally, the *L. major* infection resulted in early activation of NF-kappa-B transcription factors, followed by the up-regulation and nuclear translocation of IRF1 and IRF8^49^. In contrast, in the current study with *LmCen*^*–/–*^ infection, the expression of both IRF1 and IRF8 showed more reduction than *LmWT* infection. It has been shown that the expression of IRF1 and IRF8 may be important for IL-12 production as was shown in both *L. major* and flavivirus studies^38,56^, IRF3^−/−^IRF5^−/−^IRF7^−/−^ triple knockout mice produced minimal type-I IFNs, but a robust type-II IFN (IFN-γ) response that was stimulated by IL-12 production through IRF-1 signaling pathway^38^. This suggested that IRF1 or IRF8 may have minimal impact on type-I IFN response and that their mechanism of action is through the production of pro-inflammatory cytokine IL-12. In several bacterial infections including *Listeria monocytogenes*, *Francisella tularensis* and *Salmonella typhimurium* IRFs- 1, 3 and 5 are shown to affect the disease severity, host protection or chronicity through modulating type-I IFNs^57^. Taken together, these results suggest that among various IRFs, only the differential expression of IRF7 and its associated type-I IFNs observed in *LmCen*^*–/–*^ immunization compared to *LmWT* infection predominantly determine *LmCen*^*−/−*^ induced protective immunity.

Controlling of type-I IFN response by small molecule JAK1/JAK2 inhibitor (Ruxolitinib) following *L. donovani* infection was shown to enhance anti-parasitic CD4^+^ T cell responses by potentiating IFN-γ production^26^. The ability of *LmCen*^*–/–*^ parasites to spontaneously down-regulate IRF7 expression and correspondingly type-I IFNs indicates a novel immunoregulatory mechanism of protective immunity. In support of this argument, transient type-I IFN blockade during viral infection resulted in enhanced immunological memory. Early blockade of type-I IFNs by type-I IFN receptor blocking antibodies following infection with live attenuated viral vaccine YFV-17D and Zika virus enhanced the antigen presentation by DCs, expanded the antigen-specific CD8^+^ T cell populations and produced long-lasting immunological memory that conferred improved protection upon reinfection^58^. Even among mRNA vaccines, type-I IFN response following intradermal inoculation interfered with CD8^+^ T cell responses^59^, indicating the complex interplay between type-I and type-II IFN responses.

In conclusion this study identified a novel immunoregulatory role for type-I IFNs mainly driven by IRF7 in vaccine immunity induced by genetically modified live attenuated *Leishmania* parasites. This study builds on the previous data on the role of type-I IFNs in early and chronic stage parasitic infections and illustrates the key role of IRF-7 in orchestrating the protective immunity to *Leishmania*. Targeting IRF-7 signaling maybe more broadly applicable to anti-parasitic vaccines where controlling type-I IFNs is desirable towards controlling pathogenicity. What factors cause a spontaneous downregulation of IRF7 and IFN-α/β in *LmCen*^*−/−*^ immunization remain to be explored. Studies that showed cross-regulation of TNF-α and type-I IFNs in DCs where TNF-α was shown to suppress IFN-α production and enhance T cell activation^60^ may offer insights towards the regulation of IRF7 in *Leishmania* vaccines.

## Methods

### Mouse Infection and Immunization

Female 6- to 8-week-old C57BL/6 mice were purchased from the Jackson labs. IRF7 KO mice (B6;129P2-Irf7<tm1Ttg > /TtgRbrc) were provided by the RIKEN BRC through the National BioResource Project of the MEXT, Japan. The mice were immunized in the right ear, with either laboratory or GLP grade *LmCen*^*−/−*^ parasites (1 × 10^6^), by intradermal needle injection in the ear in 10 μl PBS using a 29 or 30-gauge needle (BD Ultra Fine). After 21 days of immunization, mice were challenged with 1 × 10^5^ total stationary phase promastigotes of *L. donovani* (LV82) intradermally by needle injection in the ear in 10 μl PBS using a 30-gauge needle. Other groups were infected with 1 × 10^6^ total stationary phase promastigotes of *L. major* WT (FV9) promastigotes in the ear in 10 μl PBS using a 30-gauge needle (BD Ultra Fine). All animals were housed in pathogen-free conditions in the vivarium at the Food and Drug Administration (FDA) animal facility, Silver Spring (MD) and the Animal Research Center, Nagasaki University. All animal experiments in this study were reviewed and approved by the Animal Care and Use Committee of the Center for Biologics Evaluation and Research, U.S. Food and Drug Administration (ASP 1995#26) and the animal care committee of Nagasaki University for ethics on animal experiments (approval number 2004271624, 2004271625, 2104011711).

### NanoString

Tissues from the inoculated ears were collected at the indicated time points into RNAlater solution. The experiments were scheduled such that the day2 and day7 post-inoculation time points fall on the same day and a common group of naïve controls could be used for normalization. Using the extracted RNA from mouse ear tissue, the Mouse Immunology V2 panel from NanoString (www.nanostring.com) was used to quantify five hundred forty-seven genes. The panel normalized mRNA transcripts using the geometric means of 14 housekeeping genes (*Alas1, Eef1g, G6pdx, Gapdh, Gusb, Hprt, Oaz1, Polr1b, Polr2a, Ppia, Rpl19, Sdha, Tbp, Tubb5*) Following hybridization and fluorescent tags, the data was analyzed by ROSALIND, Inc, (San Diego, CA). Normalization, fold changes, and *p* values were calculated using criteria provided by NanoString. The normalization of housekeeping genes was based on the geNorm algorithm. Rosalind calculation of the cell populations, distribution, heatmaps, and volcano plots followed the NanoString cell type profiling module. Similarly, the platform ROSALIND used the nCounter Advanced Analysis protocol of dividing counts within a lane using the same lane’s geometric mean of the normalizer probes. The Gene Set Analysis (GSA) was derived using the global significance score and the directed global significance score.

### Tissue collection and single cell suspension

Mice were anesthetized by isoflurane inhalation. Briefly, mice were placed in the anesthesia induction chamber with isoflurane levels of 2–2.5% with an oxygen flow rate of 1.5 L/min for 2–3 min. Complete anesthesia was verified by a firm toe pinch and confirming unresponsiveness. For recovery, mice were placed back in their cages, laying on their side with no obstructions to their nose. Euthanasia was performed by CO_2_ asphyxiation. Following euthanasia, mouse serum samples were collected in tube Serum Gel (Sarstedt cat #41.1378.005), spun at 10,000 × *g* for 5 min, then serum was transferred to new tubes and stored in –80 °C for analysis of cytokines and chemokines at the 21 days post-immunization and 14 days post-challenge experiments. Ears from immunized and challenged mice were collected. They were transferred to tubes containing RNA-Later solution (Invitrogen cat#AM7023) for 24 h. Then, the ear tissues were removed from RNA-Later and store in –80C for RNA purification (21 days post-immunization and 14 days post-challenge). From the collected spleen, and draining lymph nodes samples, in 15 ml tubes with PBS, single cell suspension was prepared, to measure cytokines. Preparation of single cell suspension entailed collecting spleen and lymph nodes into Eppendorf tubes (containing RPMI medium) in the Vivarium, placed on ice. In the lab, using about 300 µl of remaining RPMI, the lymph nodes were gently mixed using a homogenizer until a foamy layer formed. The visible fat and supernatant were removed. Sample was vortexed, 300 µl RPMI was added, and vortexed. Next, complete RPMI was added, and cells were counted. Mouse spleen samples were collected and placed RPMI within 15 ml tubes. Next, they were placed on a 70 µm cell strainer sitting on a sterile 50 mL conical tube. To prime the strainer, about (1 mL) of RPMI medium was first run through. the flat end of a sterile 3 cc syringe plunger was used to crush and mince the spleen in gentle circular motions, repeating 5 times. The cell suspension was centrifuged at 300 g for 10 min. The supernatant was removed carefully to not disrupt the pellet. Next, the tubes were vortexed to resuspend cells in the residual volume. An optional second wash was performed. Then, 3 ml ACK lysis buffer was added, and the tubes were incubated for 5 min at room temperature. Lysis was stopped by adding 5 ml of complete RPMI and centrifuged at 300 × *g* for 6 min. Cells were washed with addition of 20 ml complete RPMI medium and centrifuged again at 300 × *g* for 10 min. The cells were processed for flow cytometry analysis following antigen stimulation.

### Cell stimulation and flow cytometric analysis

The single-cell suspensions were incubated in PBS alone or PBS and with 50 µg/ml freeze-thaw *L. donovani* antigen (*LdFT*Ag) in flat bottom 48-well plates at 37 °C for 12–14 h. During last 4 h of culture, protein Transport Inhibitor (BD Golgiplug, BD BioSciences) was added to the wells. Cells were incubated with Fc-blocking (CD16/32) antibody and fixable viability dyes for 15 min at 4 °C prior to surface staining with fluorochrome-conjugated antibodies for 30 min at 4 °C (each with 1/300 dilution). Later, cells were washed with wash buffer and fixed with the Cytofix/Cytoperm Kit (BD Biosciences) for 20 min (room temperature). After fixation cells were washed two times with Perm/wash buffer (BD Biosciences). Intracellular staining was performed for 60 min at 4 °C. The complete list of antibodies is reported in (Supplementary Table 2).

Stained cells were analyzed by flow cytometry using the five-laser spectral flow cytometer Aurora (Cytek Biosciences, Fremont, CA) containing 16 violet, 14 blue, 10 yellow-green, and 8 red channels (4L-16V-14B-10YG-8R) and FACScelesta (BD bioscience). During acquisition, compensation was performed using reference controls integrated in the SpectroFlo software. Each fluorochrome peaked in individual channels, and the spectral pattern was validated based using the Cytek Full Spectrum Viewer references provided. Acquired samples were either analyzed with Spectro Flow software or were exported as FCS files and further analyzed using FlowJo software version 10.8.1 (BD Biosciences, Ashland, OR).

### Enzyme linked immunosorbent assays (ELISAs)

ELISA was used to measure IFN-α/β in serum. The level of IFN-α/β in the cell were determined by using Mouse IFN- α/β R2 ELISA kits (Cat No. EM39RB) (Invitrogen, California, USA) respectively, as per manufacturers’ protocol. Briefly, 96 welled ELISA plates (Corning Costar, Corning) were coated with 100 µl/well of diluted capture antibody in coating buffer and were incubated overnight at 4 °C. On the next day, the contents of the wells were then aspirated out and washed 4 times with 1X wash buffer (250 µl/well). Next, the wells were blocked and incubated at room temperature. After an hour, the wells were washed 4 times with 1X wash buffer. Again, 100 µl of sample was added to each well and incubated overnight at 4 °C. On the following day, the contents of the wells were washed with 1X wash buffer for 4 times. On completion, 100 µl of diluted detection antibody was added into each well and incubated for an hour followed by washing with 1X wash buffer for four times. Thereafter, 100 µl of diluted Streptavidin-HRP were then added in each well and incubated at room temperature for 45 min for IFNα/β followed by washing with 1X wash buffer for four times. Next (Chromogen Solution A and B for IRF7) and TMB for IFN-α/β were added into each well and incubated at room temperature. After 15 min of incubation, the reaction was stopped by addition of 100 µl of Stop solution. The absorption was measured at 450 nm, and wavelength. The concentrations were determined from respective linear standard curve using GraphPad Prism 9.3.1.

### Measurement of parasite burden

At 35 days post-challenge, WT C57BL/6 mice and IRF7^−/−^ mice were euthanized, and the spleen and draining lymph nodes were harvested. Single cell suspensions were prepared from the spleen and dLN samples by mechanical disruption. Serial dilution was set up The 96-well plate containing 200 μl M-199 based *Leishmania* promastigote growth medium and the plate was incubated at 27 °C. The parasite number from the twofold serial dilutions was input to GraphPad Prism for graphing and statistical analysis. Livers from infected mice were harvested, cut into small pieces, placed in C-tubes containing 3 mL of enzymatic digestion mix (Collagenase Type I, 0.1%, and DNase I, 0.05% in M199 media). The liver tissues were homogenized to single cell suspension using gentle MACS according to manufacturer’s protocol. Enzyme activity was stopped by adding EDTA on ice. Cells were washed twice with complete M199 media and resuspended in 2 mL of complete media. Finally, a serial dilution of liver cells was performed in 96-well plates. After 12–15 days of incubation at 26 °C, parasite growth in each well was examined using the inverted microscope.

### Statistics

Enrichment was calculated relative to a set of background genes relevant for the experiment, using Cytoscape (Version 3.9.1). Data analyzed in ROSALIND was downloaded. The differential expression data of volcano plots was plotted using the −log10 (*p* value) and log2 fold change. Comparisons were made between treatments using GraphPad Prism (Version 9.3.1), (GraphPad Software, San Diego, CA) using two-way ANOVA or *t* test, depending on the study or graph.

Statistical analysis of differences between means of groups was determined by unpaired two-tailed Mann–Whitney *t* test. A *p* value < 0.05 was considered significant (*), and *p* values < 0.005 (**) or <0.001 (***) were considered highly significant.

## Supplementary information

Supplementary material

## Data Availability

Raw data from the Nanostring experiments obtained as. RCC files has been uploaded in GEO and is accessible in the link below. https://www.ncbi.nlm.nih.gov/geo/query/acc.cgi?acc=GSE230929.
