# Supplementary material for: Downregulation of IRF7-mediated type-I interferon response by LmCen–/– parasites is necessary for protective immunity
Source: NPJ Vaccines. 2024 Dec 19;9:250. doi: 10.1038/s41541-024-01032-6 (PMC11659581; doi:10.1038/s41541-024-01032-6)
Supplement: Supplementary file 1 — Supplementary material [file 41541_2024_1032_MOESM1_ESM.pdf]

Supplementary Table 1: Top 10 upregulated and downregulated genes

(A) *LmCen*$^{-/-}$ vs Naïve at 2 days p.i.

	Fold Change	p-Value	p-Adj
Clec4e	5.70966	1.59E-05	1.90E-03
Cxcl10	5.17393	3.55E-04	1.30E-02
Il1b	4.91089	2.96E-05	2.61E-03
S100a8	4.89051	4.60E-04	1.33E-02
Fcgr1	4.51383	1.41E-05	1.90E-03
Fcgr4	4.1964	3.97E-03	5.79E-02
S100a9	3.81831	1.13E-03	2.66E-02
Gzma	3.41504	3.10E-02	1.62E-01
Ccr5	2.80116	5.26E-03	6.61E-02
Irf7	2.71749	1.37E-03	3.00E-02
C7	-1.5025	1.15E-03	2.66E-02
Cfd	-1.16364	3.92E-02	1.82E-01
H2-Ob	-0.917538	3.13E-02	1.62E-01
Icam4	-0.87193	5.61E-03	6.67E-02
Lef1	-0.716522	1.75E-03	3.12E-02
Pdgfb	-0.684092	1.77E-03	3.12E-02
Tgfb2	-0.650224	4.10E-02	1.86E-01
Src	-0.618533	7.74E-03	8.51E-02
Ifngr2	-0.535719	3.53E-02	1.76E-01
Phlpp1	-0.501266	1.87E-02	1.34E-01

(B) *LmCen*$^{-/-}$ vs Naïve at 7 days p.i.

	Fold Change	p-Value	p-Adj
Cxcl10	8.15406	6.00E-06	7.45E-04
Sell	7.75622	1.54E-05	9.31E-04
Gzma	7.72564	1.02E-03	1.08E-02
Fcgr4	7.24238	6.79E-05	2.49E-03
Cxcl9	6.38946	2.69E-03	1.69E-02
Gzmb	6.32797	3.66E-03	2.01E-02
Clec4e	5.60979	1.91E-05	9.31E-04
Il1b	5.50117	8.23E-06	7.45E-04
Fcgr1	4.87176	6.58E-06	7.45E-04
Ccr5	4.54411	1.56E-04	4.10E-03
Tnfsf18	-1.53605	2.01E-03	1.38E-02
Cfd	-1.46346	1.00E-02	3.62E-02
Kir3dl2	-1.39046	1.88E-02	5.62E-02
Xcl1	-1.19391	5.28E-04	7.75E-03
Abcb1a	-1.04423	2.23E-04	5.45E-03
Icam4	-1.00868	1.33E-03	1.14E-02
Rorc	-0.91716	4.03E-05	1.61E-03
Cd36	-0.895481	8.76E-03	3.38E-02
Vtn	-0.823122	4.22E-03	2.21E-02
Cd55	-0.76776	1.68E-03	1.25E-02

(C) *LmWT* vs Naïve at 2 days p.i.

	Fold Change	p-Value	p-Adj
S100a8	6.02745	2.87E-05	2.62E-03
Sell	5.46597	3.29E-04	1.08E-02
Clec4e	5.46489	2.44E-05	2.62E-03
S100a9	5.12387	3.57E-05	2.62E-03
Il1b	5.03709	2.34E-05	2.62E-03
Cxcl10	4.54535	7.48E-04	1.57E-02
Fcgr4	4.24159	3.74E-03	3.68E-02
Fcgr1	4.04755	3.00E-05	2.62E-03
Gzma	3.152	4.22E-02	1.53E-01
Cxcl1	2.65711	1.35E-02	7.69E-02
C6	-2.19114	3.63E-03	3.68E-02
Kir3dl2	-1.31451	2.63E-02	1.18E-01
Tgfb2	-1.18925	1.45E-04	7.11E-03
Fcamr	-1.10362	3.07E-03	3.56E-02
Irf4	-1.00922	3.92E-04	1.08E-02
Tnfsf12	-0.989062	4.99E-03	4.22E-02
Ctsg	-0.952694	1.31E-02	7.68E-02
H2-Ob	-0.878009	3.96E-02	1.48E-01
Mapk11	-0.865699	3.67E-03	3.68E-02
Il18r1	-0.832195	2.84E-02	1.24E-01

(D) *LmWT* vs Naïve at 7 days p.i.

	Fold Change	p-Value	p-Adj
Gzma	9.07206	1.38E-05	5.37E-04
Cxcl10	8.64169	1.42E-06	2.97E-04
Sell	8.46842	2.34E-06	2.97E-04
Fcgr4	8.19311	5.74E-06	4.21E-04
Gzmb	6.61723	2.07E-03	8.30E-03
Clec4e	6.40939	2.70E-06	2.97E-04
Cxcl9	5.74269	9.66E-03	2.47E-02
Fcgr1	5.66804	2.77E-07	1.22E-04
Il1b	5.57238	6.76E-06	4.25E-04
Ccl5	5.29278	1.60E-04	2.08E-03
Icam4	-0.924623	3.40E-03	1.14E-02
Cd36	-0.802796	2.23E-02	4.63E-02
Pdgfb	-0.676071	1.96E-03	7.96E-03
Il22ra2	-0.649646	1.11E-02	2.75E-02
Vtn	-0.616671	3.13E-02	6.00E-02
Rorc	-0.607065	9.36E-03	2.41E-02
Mbp	-0.604974	3.71E-06	3.26E-04
Tlr5	-0.56309	3.03E-03	1.08E-02
Defb1	-0.555831	2.11E-02	4.46E-02
Pparg	-0.536053	1.15E-02	2.78E-02

Supplementary Table 1: **Fold change values of the top differentially expressed genes from the transcriptomic analysis using NanoString.** Top 10 significantly upregulated genes and downregulated genes in LmCen$^{-/-}$ vs Naive at (**A**) 2 days and (**B**) 7days post immunization. Similarly (**C-D**) showing Top 10 upregulated and downregulated genes among LmWT vs Naïve at (**C**) 2days and (**D**) 7days post injection, ranked by log FC. P-value and P-adjusted for false discovery rate (fdr) are also included.

Supplementary Table 2: Reagents and Resources

REAGENT or RESOURCE	SOURCE	IDENTIFIER
**Antibodies**		
APC anti-mouse CD45, clone QA17A26	Biolegend	Cat#157606, 100µg
APC-Cyanine7 anti-mouse CD45, clone QA17A26	Biolegend	Cat#157618, 100µg
Alexa Flour 700 anti-mouse CD3, clone 17A2	Biolegend	Cat#100215, 25µg
Alexa Fluor™ 488 anti-mouse CD3e, clone 145-2C11	eBioscience	Cat#53-0031-82, 100µg
BV650 anti-mouse CD4, clone GK1.5	Biolegend	Cat#100469, 50µg
BV786 Rat Anti-Mouse CD4, clone GK1.5	BD Biosciences	Cat#563331, 50µg
APC/Cyanine7 anti-mouse CD8a Antibody	Biolegend	Cat#100713, 25µg
BV650™ anti-mouse CD8a, clone 53-6.7	Biolegend	Cat#100742, 50µg
FITC anti-mouse/human CD44, clone IM7	Biolegend	Cat#103005, 50µg
PerCP-Cyanine5.5 anti-mouse/human CD44, clone IM7	Biolegend	Cat#103031, 25µg
PE/Cyanine7 anti-mouse IFN-γ, clone XMG1.2	Biolegend	Cat#505825, 25µg
PE anti-mouse IFN-γ, clone XMG1.2	eBioscience	Cat#17-73311-81, 50µg
BV605 anti-mouse TNF-α, clone MP6-XT22	Biolegend	Cat#506329, 125µg
PE-Dazzle 594 anti-mouse TNF-α, clone MP6-XT22	Biolegend	Cat#506345, 25µg
BV711 anti-mouse IL-2 clone JES6-5H4	Biolegend	Cat#503837, 50µg
APC anti-mouse IL-2 clone JES6-5H4	eBioscience	Cat#17-7021-81, 50µg
PE anti-mouse IL-10, clone JES5-16E3	Biolegend	Cat#505007, 25µg
BV421 Anti-mouse IL-10, clone JES5-16E3	Biolegend	Cat# 505021, 25µg
**Chemicals**		
LIVE/DEAD™ Fixable Blue Dead Cell Stain Kit	eBioscience	Cat#L23105
Zombie Aqua™ Fixable Viability Kit	Biolegend	Cat# 423101
Brefeldin A Solution (1,000X)	Biolegend	Cat# 420601
Protein Transport Inhibitor (Containing Brefeldin A)	BD Biosciences	Cat#555029
Cell Activation Cocktail (without Brefeldin A)	Biolegend	Cat#423301
BD Leukocyte Activation Cocktail, with BD GolgiPlug	BD Biosciences	Cat#550583
Cell Stimulation Cocktail (500X)	eBioscience	Cat#00-4970, (4 x 100µg)
TheraPEAKTM ACK Lysing Buffer (1x)	Lonza	Cat#BP10-548E
Ficoll® PM 400	Sigma	Cat# F4375-100G
Water-deionized & sterilized	Nacalai Tesque Inc.	Cat# 06442-95
RPMI-1640 Medium	Sigma	Cat# R8758-500ML
Fetal Bovine Serum	Sigma	Cat# F2442
Penicillin-Streptomycin (100x)	Wako	Cat# 168-23191
D-PBS (-)	Wako	Cat# 045-29795
Cell Staining Buffer	Biolegend	Cat# 420201- 500ML
Cyto-Last™ Buffer	Biolegend	Cat# 422501- 500ML
BD Cytofix/Cytoperm™ Solution	BD Biosciences	Cat# BDB554722
BD Perm/Wash™ Buffer	BD Biosciences	Cat# BDB554723
**Critical commercial assays**		
nCounter XTCodeSet_XT_PGX_MmV1_Immunology	NanoString Technologies	115000052 (Lot#RC3788X1)
nCounter Master Kit Cartridge	NanoString Technologies	100052 (Lot#050821)
nCounter Master Kit Prep Plate	NanoString Technologies	100052 (Lot#160719)
nCounter Master Kit Prep Pack	NanoString Technologies	100052 (Lot#170821)
Micro sample tube Serum Gel, 1.1 ml, screw cap, EU/ISO	Sarstedt	Cat#41.1378.005
RNeasy Protect Animal Blood Kit (50)	QIAGEN	Cat#73224
RNAprotect Animal Blood Tubes (100 µl) Stabilization reagent for 100 µl blood	QIAGEN	Cat#76544
Mouse interferon regulatory factor 7 (IRF7) ELISA Kit	MyBioSource	Cat#MBS2610968
Mouse IFN-Alpha/Beta R2 ELISA	Invitrogen	Cat# EM39RB
DuoSet Mouse TNF-α ELISA Kit	RD system	Cat# DY410
DuoSet Mouse IFN-γ ELISA Kit	RD system	Cat# DY485
**Software and algorithms**		
FlowJo v10.8.1 Software	BD Life Sciences	www.flowjo.com
FACS Diva (v8.0.2)	BD Biosciences	www.bdbiosciences.com
ClustVis	BIIT	www.biit.cs.ut.ee/clustvis/
GraphPad Prism (v9.4.1)	GraphPad Software Inc	www.graphpad.com
Cytoscape (Version 3.9.1)	Cytoscape Consortium	www.cytoscape.com
ROSALIND	Rosalind Inc.	www.rosalind.onramp.bio/

LmCen$^{-/-}$ vs Naïve 7 days p.i.

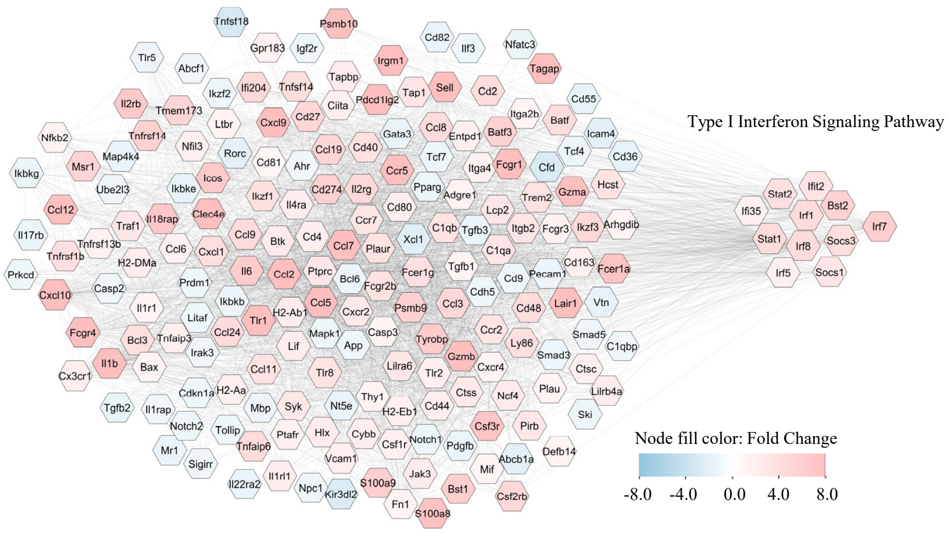

*Lm*WT vs Naïve 2 Days p.i

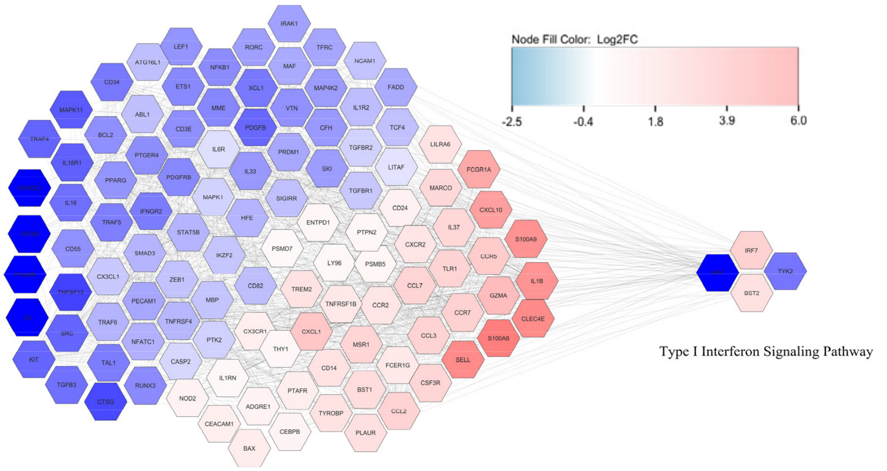

*Lm*WT vs Naïve 7 Days p.i.

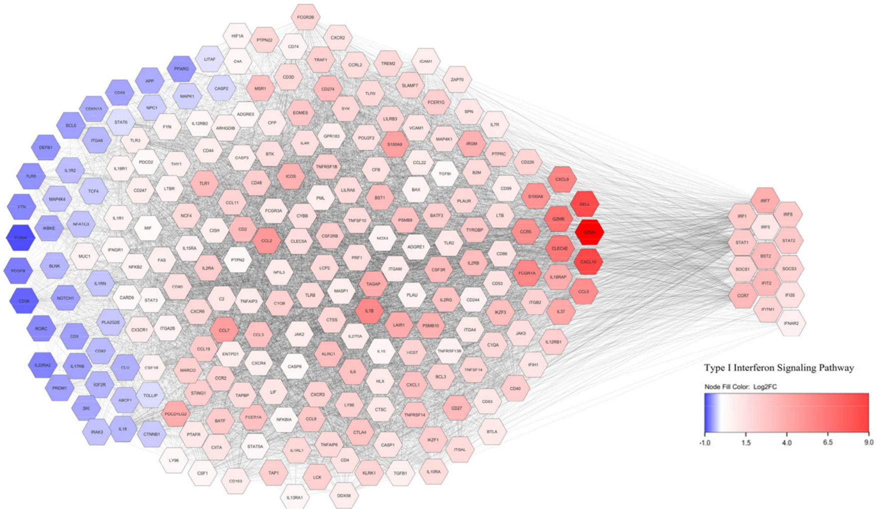

Supplementary Figure 1: Distinct transcriptional profiles in tissues of C57BL/6 mice immunized with *LmCen*$^{-/-}$ or infected with *LmWT* parasites. (A) The gene network through GO enrichment analysis of *LmCen*$^{-/-}$ immunized vs Naïve at 7 days post immunization, **(B)** LmWT infected vs Naïve at 2 days and (C) 7 days post-infection showing the upregulation of transcripts associated with the IRF7 mediated type I IFN response in the ear tissues.

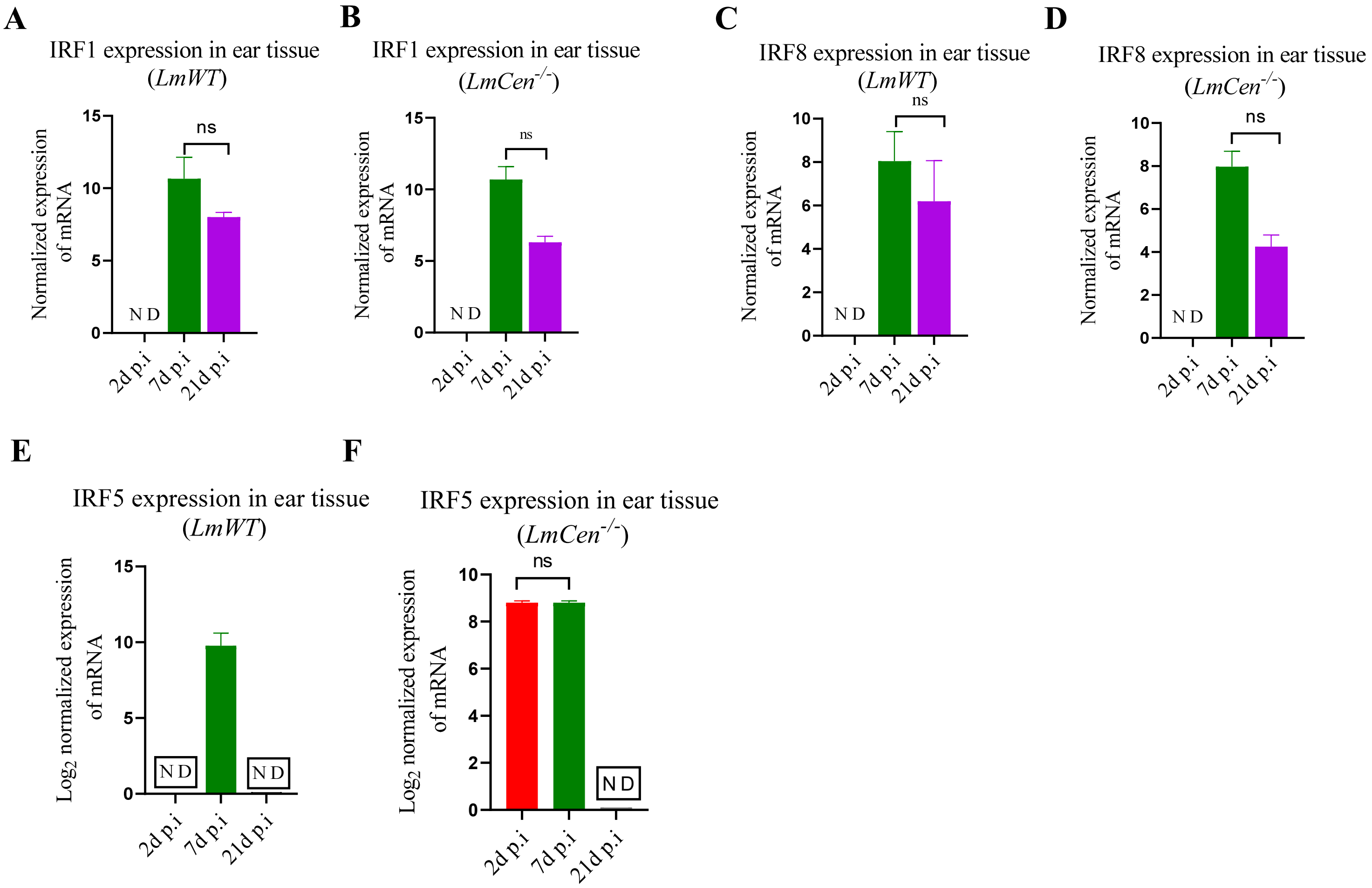

Supplementary Figure 2: **IRF1 and IRF8 expression in C57BL/6 mice immunized with *LmCen$^{-/-}$* parasites at 2 days, 7 days and 21 days post-immunization.** Normalized expression of IRF1 measured at the site of inoculation (ear tissue) by NanoString between 2 dyas, 7 days and 21 days p.i in (**A**) *LmWT* and (**B**) *LmCen$^{-/-}$*, IRF8 expression between 7 days and 21 days p.i in (**C**) *LmWT* and (**D**) *LmCen$^{-/-}$* inoculation, and IRF5 expression in (**E**) *LmWT* and (**F**) *LmCen$^{-/-}$*. Results are shown as mean ± SD representing one experiment and p-value determined with Mann-Whitney two-tailed test.

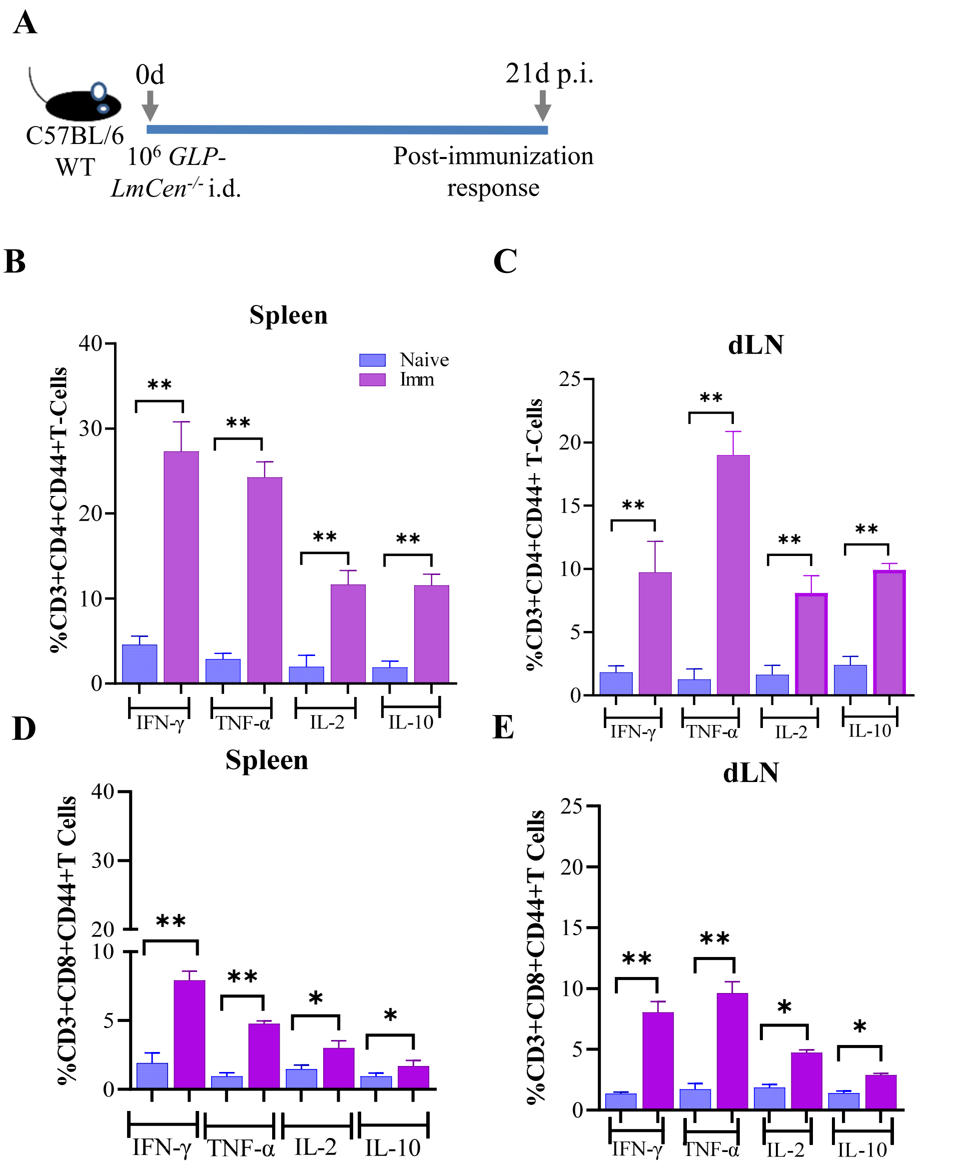

Supplementary Fig 3: Host protective Th1 immune response following immunization with *LmCen*$^{-/-}$ parasites in C57BL/6 mice. (A) Schematic representation of the experimental study conducted at the FDA laboratory. Production of cytokines IFN-γ, TNF-α, IL-2, and IL-10 was measured in CD4^{+} T cells isolated from spleen and stimulated with *Leishmania donovani* antigens at 21 days post immunization (**B**). Similar response from the CD4^{+} T cells isolated from the draining lymph nodes is shown (**C**). Cytokine expression from the CD8^{+} T cells isolated from the spleen (**D**) and from draining lymph nodes (**E**) is shown. Results (the geometric means with 95% Cl) represent one experiment with the p-values determined by Mann-Whitney two-tailed test.

A

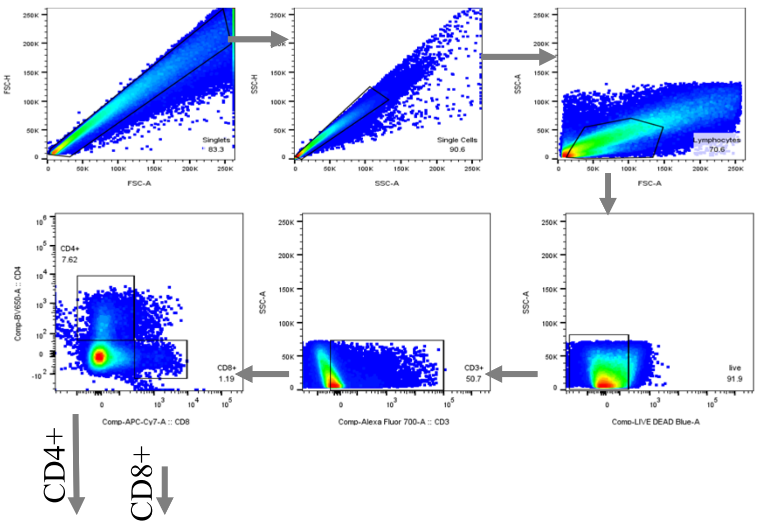

B

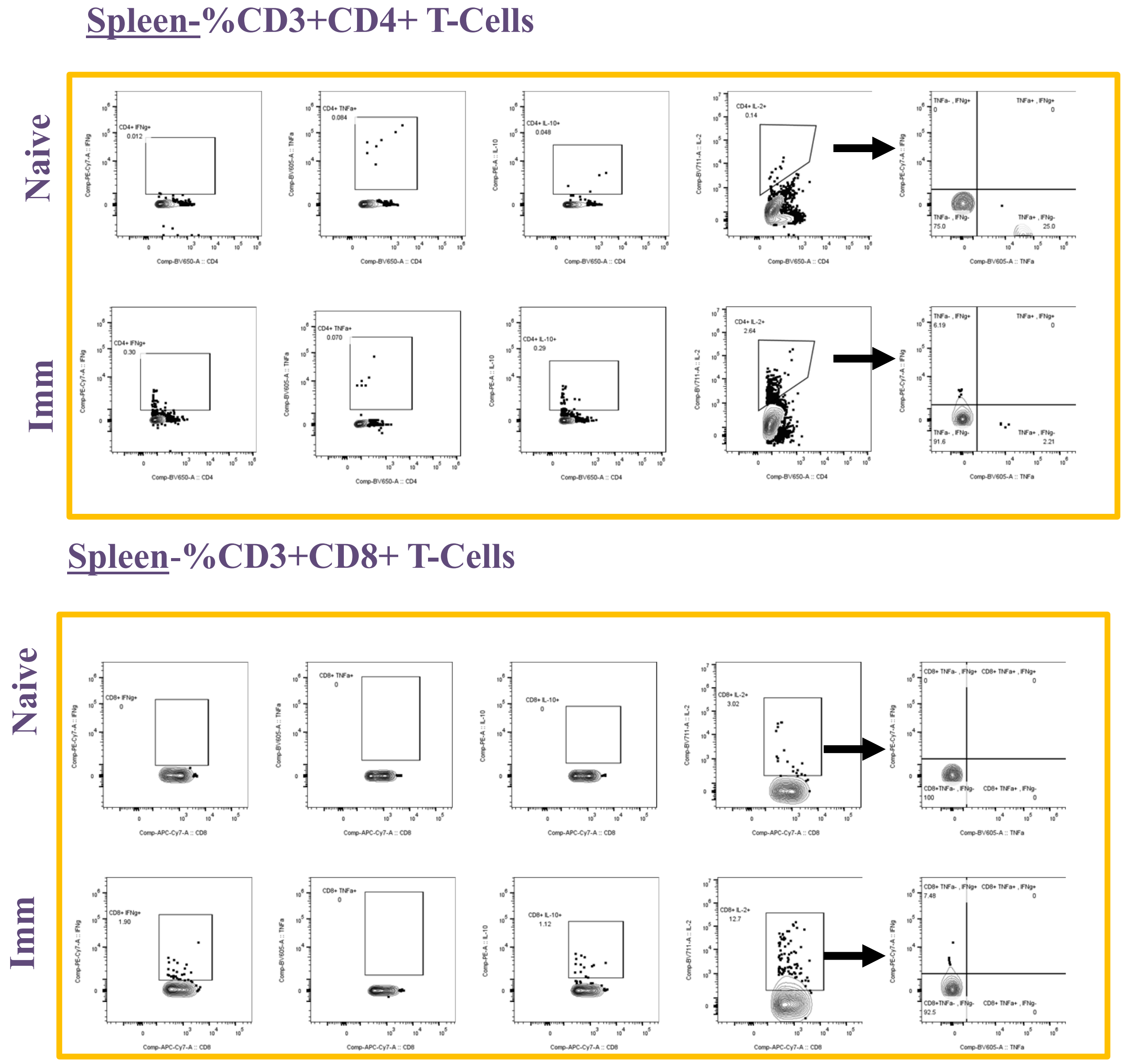

Supplementary Figure 4: **Gating strategy for identifying specific T cell populations in WT mice at 21 days post-immunization and 14 days post-challenge performed at FDA. A)** Multiparameter flow-cytometry based analysis for cytokine secreting T cells from naïve, *LmCen*$^{-/-}$ immunized mice. **B)** Multiparameter analysis for multiple cytokine secreting live $CD3^{+}CD4^{+}$ T cells and $CD3^{+}$ $CD8^{+}$ T cells after 24 h of in-vitro re-stimulation with freeze-thaw *L. donovani* antigen (*LdFT*Ag) from spleen or draining lymph nodes of C57Bl/6 mice.

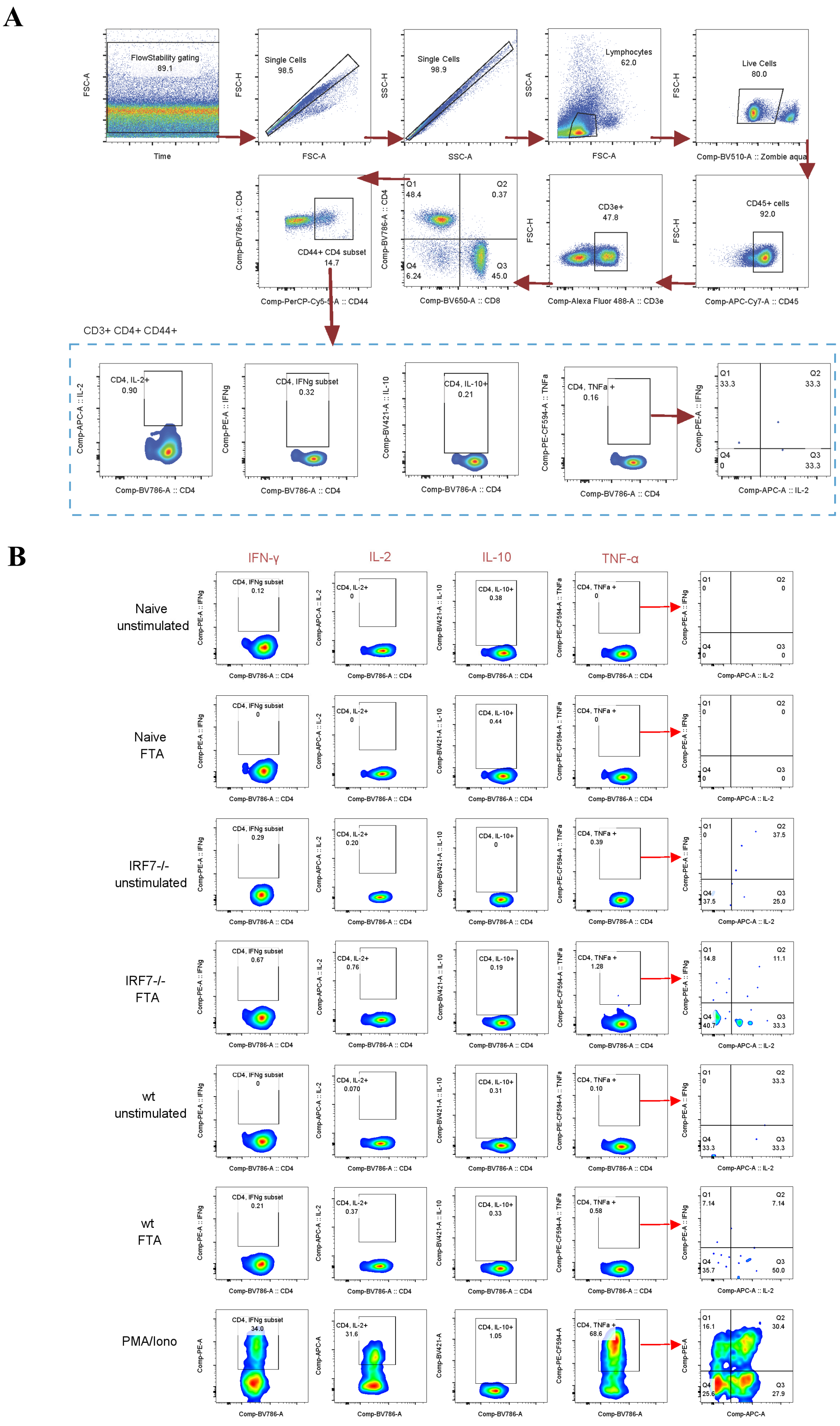

Supplementary Figure 5: **Gating strategy for identifying specific T cell populations in WT and IRF7$^{-/-}$ mice at 21 days post-immunization and 14 days post-challenge performed at Nagasaki University. A)** Multiparameter flow-cytometry based analysis for cytokine secreting T cells from naïve, *LmCen*$^{-/-}$ immunized mice. **B)** Multiparameter analysis for multiple cytokine secreting live CD3^{+}CD4^{+}CD44^{+} T cells after 24 h of in-vitro re-stimulation with freeze-thaw *L. donovani* antigen (*LdFT*Ag) from spleen or draining lymph nodes of C57Bl/6 or IRF7$^{-/-}$ mice.

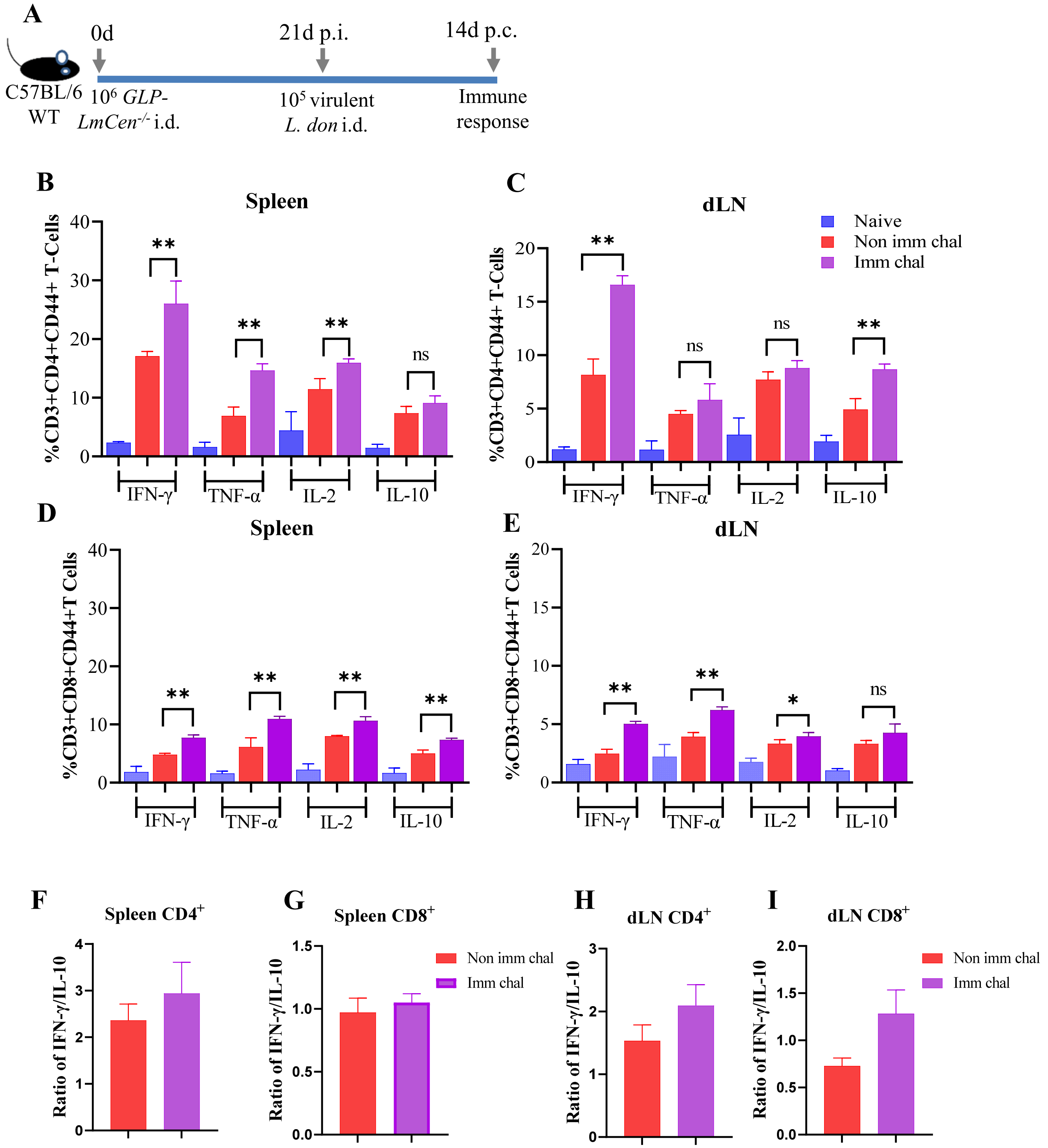

Supplementary Fig 6: Host protective Th1 response following challenge with virulent *L. donovani* in *LmCen*$^{-/-}$ immunized C57BL/6 mice . (**A**) Schematic representation of the experimental study. Measurement of IFN-γ, TNF-α, IL-2, and IL-10 levels from activated CD4^{+}T cells **(B)** and from activated CD8^{+} T cells **(D)** from spleen at 14 days post challenge. Measurement of IFN-γ, TNF-α, IL-2, and IL-10 levels from activated CD4^{+} T cells **(C)** and from activated CD8^{+} T cells **(E)** from draining lymph nodes at 14 days post challenge. The ratio of IFN-γ/IL-10 expression in the spleen for CD4^{+} T cells **(F)** and CD8^{+} T cells **(G)**. The ratio of IFN-γ/IL-10 expression in CD4^{+} T cells **(H)** and CD8^{+} T cells **(I)** isolated from the draining lymph node. Results (the geometric means with 95% Cl) represent one experiment with the p-values determined by Mann-Whitney two-tailed test.

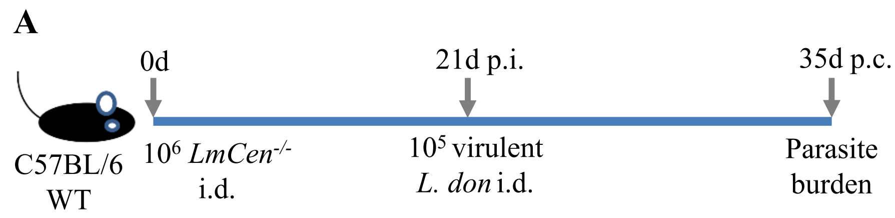

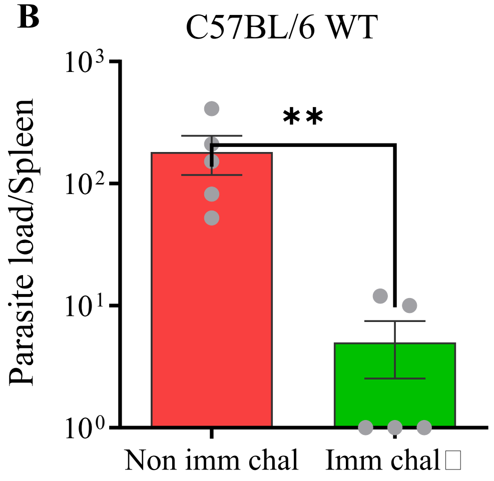

Supplementary Fig 7: Splenic parasite burden in control and IRF7$^{-/-}$ mice: (A) Schematic representation of the experimental study. **(B)** Splenic parasite burden of *LmCen*$^{-/-}$ immunized (Imm Chal, n=5) and age-matched non-immunized (Non-imm Chal, n=5) C57BL/6 mice were determined at 5 weeks post needle challenge with virulent *L. donovani* parasites. Results are shown as mean ± SD representing one experiment and p-value determined with Mann-Whitney two-tailed test.
